# The interrelationship between meteorological parameters and leptospirosis incidence in Hambantota district, Sri Lanka 2008–2017 and practical implications

**DOI:** 10.1371/journal.pone.0245366

**Published:** 2021-01-22

**Authors:** N. D. B. Ehelepola, Kusalika Ariyaratne, D. S. Dissanayake

**Affiliations:** 1 The Teaching (General) Hospital–Peradeniya, Peradeniya, Sri Lanka; 2 Lanka Hydraulic Institute, Moratuwa, Sri Lanka; 3 Faculty of Medicine, University of Peradeniya, Peradeniya, Sri Lanka; University of Minnesota, UNITED STATES

## Abstract

**Background:**

Leptospirosis is a bacterial zoonosis. Leptospirosis incidence (LI) in Sri Lanka is high. Infected animals pass leptospires to the environment with their urine. Leprospires' survival in the environment to infect a new host depends on meteorological factors. El Nino Southern Oscillation (ENSO) and Indian Ocean Dipole (IOD) modulate the weather in Sri Lanka.

**Objectives:**

The determination of interrelationship between the LI in the Hambantota District, and local meteorological parameters, ENSO and IOD.

**Methods:**

We acquired notified leptospirosis cases in the Hambantota District and population data. We calculated weekly leptospirosis incidences for 2008 to 2017.Weather data from two weather stations was obtained, averaged and converted into weekly data. We plotted time series graphs and observed the correlation between seven aggregated weather parameters and LI. We estimated cross-correlations between those weather parameters and LI. As our principal analysis we determined correlation between LI and seven local weather parameters, Nino 3.4, Nino4 and Dipole Mode Index (DMI) indices using wavelet analysis.

**Results:**

Our wavelet analysis results showed troughs of minimum, maximum, mean temperatures, soil temperature, the evaporation rate, the duration of sunshine were followed by peaks in LI and peaks of rainfall followed by peaks of LI, all after lag periods. Our time series graphs and cross-correlation determination results are generally in agreement with these results. However there was no significant correlation between rainfall and LI in the cross-correlation analysis. There were peaks of LI following both peaks and troughs of DMI. There was no clear correlation between both Nino indices and LI.

**Discussion:**

This may be the first long-term study demonstrating soil temperature, evaporation rate and IOD are correlating with LI. The correlation pattern of LI with temperature parameters differs from similar past studies and we explain the reasons. We propose ways to control high LI we observed after periods of weather favorable for transmission of leptospirosis.

## 1. Introduction

Leptospirosis is an emerging spirochaetal zoonosis of worldwide distribution but more prevalent in countries with hot and wet climates like in Sri Lanka. Leptospirosis is a major public health problem of people and domestic animals [1–5]. It is an endemic disease of Sri Lanka, and has been a notifiable disease since 1991 [3,6,7]. Leptospirosis is treatable but severe leptospirosis (Weil's disease) results in severe morbidity and high mortality [1,3,5,6].

### 1.1 The pathogen

Leptospirosis is caused by infection with pathogenic bacteria of genus *Leptospira*. These are classified in to >300 serovars arranged in 25 sero groups based on their antigenic properties and by some 14 species based on their DNA-relatedness [1–3,8,9]. Six primarily soil-living *intermediate species of Leptospira can also cause human disease opportunistically [9]. Five species of Leptospira have been isolated from Sri Lankans in the past and 15 serogroups have been reported [9].*

### 1.2 Life cycle of the pathogen

Synanthropic rodents and domestic mammals are the main natural hosts of pathogenic leptospira and humans are incidental hosts [1–4,6,10]. Infected hosts pass leptospires to the environment mainly with their urine. Humans commonly get infected by leptospires from damp soil, surface water. It is seldom by direct contact with infected animals [1–4,6,11,12]. *Leptospires enter the body via breaches of the skin or through a mucus membranes. Intact mucus membranes also be a cause of leptospirosis [1–3].*

### 1.3 How meteorological parameters influence the life cycle of the pathogen

Soil moisture and temperature, presence of surface water, humidity and solar radiation are reported to influence survival of leptospires, their ability to form biofilms in the environment until they enter a host’s body [1–4,11,13]. Biofilms help a leptospire load that is sufficient enough to cause infection, enter in to the host’s body. Desiccation rapidly kills leptospires [10]. These factors, which control the survival of leptospires in the environment, depend on local weather. Therefore, the leptospirosis transmission is influenced by local weather [1–4,6,11]. The pest rodent population size of a locality (in the tropics) and the activity of rodents, fraction of leptospirosis-infected rodents are known to be influenced by the local weather [7,11,13–16]. The two traditional cultivation seasons (especially rice cultivation) of Sri Lanka named ‘Yala’ and ‘Maha’ (called ‘Kharif’ and ‘Rabi’ in other South Asian countries) are based upon the two major monsoon seasons of the year [6,7]. People who cultivate certain crops especially those who work in rice paddies in Sri Lanka are at risk of contracting leptospirosis due to the following reasons [1,3,6,11,12,17]. Although going out of practice, water buffaloes and cattle are used in rice field preparation and threshing of harvest in SriLanka and people come into close contact with their urine during those activities. Most people work in rice paddies barefoot and barehanded and after working wash themselves and their buffaloes and cattle in nearby water channel cascades that irrigate paddies. Those channels act as reservoirs and a mode of spread of leptospires [6,12]. Animal (especially cattle) manure is a common traditional fertilizer used by Sri Lankan farmers and it is usually applied to crops without wearing any gloves [6]. Cattle manure was demonstrated to be a source of pathogenic leptospires in field conditions [13]. Small mammals, including rodents, are part of the rice field ecosystem of Sri Lanka [18]. Considering all those influences on the life cycle of pathogenic leptospires by meteorological parameters, there are good reasons to presume that meteorogical parameters have an important influence on leptospirosis transmission in Sri Lanka, thus local leptospirosis incidence [6].

### 1.4 Reasons to study the correlation between LI and coupled ocean atmosphere phenomena

El Nino southern oscillation (ENSO) is a coupled ocean atmosphere phenomenon where changes of sea surface temperature (SST) in the Eastern and Western sides of the equatorial Pacific Ocean and coupled atmospheric pressure oscillations modulate the weather in many parts of the world, including Sri Lanka [19]. Indian Ocean dipole (IOD) is the comparable coupled ocean atmosphere phenomena related to the tropical Indian Ocean. IOD is also known to influence weather in Sri Lanka [19,20]. Hence both ENSO and IOD are likely to modulate LI in Sri Lanka. To the best of our knowledge there are no long term past studies regarding IOD’s influence on LI from anywhere and on ENSO’s influence from Sri Lanka. Thus additionally we decided to study the correlation between indices of ENSO, IOD versus leptospirosis incidence of Hambantota district as well. There are several indices of ENSO. Those are different computations of SST and atmospheric pressure anomalies between different locations of the Eastern and Western sides of the tropical Pacific Ocean. Nino3.4 and Nino4 are computed considering areas closer to Sri Lanka. Because both indices were selected for and shown to be correlated with the weather of Sri Lanka by some past studies, we selected both those indices for our study [19–21].

### 1.5 Motives for this study

Compared to the significance of the burden of leptospirosis, long duration studies on leptospirosis-weather interrelationship are rare [6,8]. Only a few long term studies in the past worldwide have studied how multiple meteorological parameters correlate with the leptospirosis incidence of an area [1,4,6,8]. We did not find any studies that consider soil temperature and evaporation rate (even though some past studies considered atmospheric temperature). The effects of the duration of sunshine on leptospirosis incidence were never studied in Sri Lanka according to the best of our knowledge. Considering this background, we have decided to study the interrelationship between leptospirosis incidence and rainfall, soil temperature, air temperature, evaporation rate and the duration of sunshine in the Hambantota district in Sri Lanka, where a higher percentage of the working population is engaged in agriculture, especially rice cultivation, compared to most other districts in the country.

### 1.6 Objectives and hypotheses

Our objective was to determine the interrelationship between weekly leptospirosis incidence and the following meteorological parameters of the Hambantota district for the period of 2008–2017 and to identify potential ways to use the information generated to improve the control of local leptospirosis. Those weather parameters are: weekly averages of rainfall, maximum, mean and minimum atmospheric temperature, evening soil temperature at 5cm, evaporation rate and hours of sunshine.

Detecting any interrelationship between the two monthly indices of ENSO (Nino 3.4 and Nino 4 SST anomaly indices) plus the monthly IOD SST anomaly index namely, Dipole Mode Index (DMI) versus the monthly leptospirosis incidence of Hambantota was an objective as well.

## 2. Methods

### 2.1 Ethics statement

The study was approved by the ethics review committee of the Faculty of Medicine, Peradeniya University of Sri Lanka (2018/EC/22).

### 2.2 Study setting

**Fig 1:**
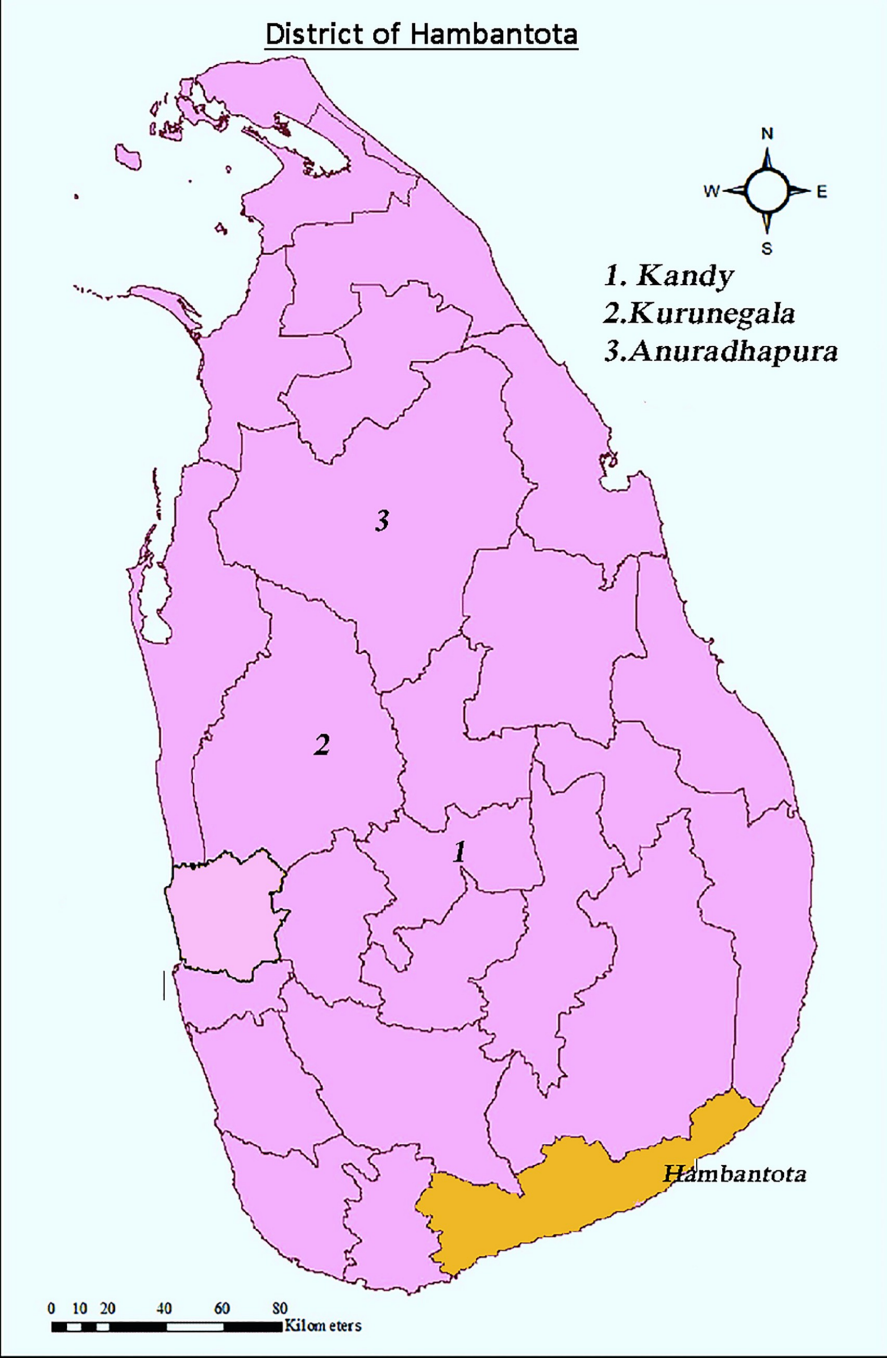
Hambantota district (the area of study) in a map of Sri Lanka. Hambantota district is shaded in yellow color in contrast to the other 24 districts. Kandy, Anuradhapura and Kurunegala districts that we mention in our discussion are also identified in this map. This map is a modification of the map (https://journals.plos.org/plosone/article?id=10.1371/journal.pone.0231408) created under CC BY4.0.

Hambantota district has an area of 2,593km^2^. Hambantota town is situated in the middle of the district. The estimated population of the district in 2017 was 647000. Among the working population, 43% and 32.2% respectively were engaged in agriculture in 2010 and 2017, according to the Sri Lanka Department of Census and Statistics. To compensate for local differences of weather within the district as well as to compensate for when data is missing from one station, we used averages of weather data from two weather stations of the district, namely Angunakolapelessa (6.17N,80.88E) and Weerawila (6.28N,81.23E) for our study. Fig 1 shows the Hambantota district in a map of Sri Lanka.

To give an idea of the local weather to the readers, we did the following estimates for our study period. The average annual rainfall was 1175mm, average mean ambient temperature was 28.3 degrees Celsius, average soil temperature at 5cm depth in evenings was 34.6 degrees Celsius, average evaporation rate was 4.3mm/day and average hours of sunshine per day was 6.9 hours.

### 2.3 Data

We obtained the numbers (counts) of leptospirosis cases notified from our study area each week from the weekly epidemiology reports of the Ministry of Health of Sri Lanka from 2008 to 2017 for this retrospective analytical study. We acquired the rainfall, atmospheric and soil temperature, evaporation rates and sunshine hours data of Angunakolapelessa and Weerawila weather stations from Sri Lanka Department of Meteorology for the same period. The annual estimated mid-year population of the Hambantota district for 2008–2017 was obtained from the Sri Lanka Department of Census and Statistics. Monthly Nino 3.4 and Nino 4 SST anomaly indices, plus monthly IOD SST anomaly index known as dipole mode index (DMI) were obtained online from the National Oceanic and Atmospheric Administration of the United States. Our data sharing statement gives further details. For weeks when data was missing from one weather station, only the data of the other weather station was considered.

### 2.4 Analysis

We have estimated weekly leptospirosis incidence (LI) per 100,000 population. We plotted time series graphs and observed the correlation patterns between seven aggregated weather parameters and leptospirosis incidence during the course of the 52 weeks of the year, between 2008–2017. As there are clear annual cycles of weather in Sri Lanka, this helps us get a basic idea of LI change with the changes of meteorological parameters within a year. The total count of reported cases in 2011(428) was approximately the same as the combined total of the next four years (441). To find any special weather condition contributing to this high LI we plotted time series graphs and compared aggregated weather parameters of the other nine years against 2011.

We used wavelet time series analysis (wavelet analysis) as our mainstay analysis method. Wavelet analysis is one of the best methods for the determination of nonlinear and non-stationary correlations like those between weather parameters and LI. It has been used widely in the recent past in ecology, and in particular to determine correlations between weather and infectious diseases [22,23]. However, we could not determine the scale of the correlations with that method [6]. To determine which weather parameter/s most strongly affects LI of Hambantota we have to find the magnitudes of the correlations. We computed cross-correlation coefficients between weather parameters and LI for that. We used weekly data to analyze weather parameters and then converted leptospirosis incidence to monthly data and determined correlations between monthly Nino3.4, Nino4 and DMI indices.

#### 2.4.1 Wavelet analysis

The same methodology has been used by us in two studies in the past and explained in detail in the methodology sections of those papers distributed under CCA BY 4.0 and available online [6,23].

Most mathematical methods that examine periodicities in the frequency domain, such as the Fourier analysis, implicitly assume that the process is stationary in time. However, wavelet transforms expand time series into time-frequency space and can therefore find localized intermittent periodicities.

In wavelet analysis, a suitable window is chosen and this window is shifted along the signal and for every position the spectrum is calculated. This procedure is repeated numerous times with a slightly shorter and longer window for every new cycle. With wavelet transform the outcome will be a collection of time-frequency representations of the signal with different resolutions.

The significance of the wavelet transform lies in the fact that it can be used to analyze time series that contain non-stationary power at diverse frequencies. By decomposing a time series into time-frequency space, we can determine both the dominant modes of variability and how those modes vary in time. Cross wavelet transform (XWT) and wavelet coherence (WTC) are used for examining the correlation in time frequency space between two time series.

While XWT is a common tool for studying localized intermittent oscillations in a time series, it is very often appropriate to examine two time series together that may be expected to be linked. In particular, to examine whether regions in time frequency space with large common powers have a consistent phase relationship and are therefore suggestive of causality between those time series.

Continuous wavelet transform (CWT) was determined for the meteorological variable we studied. The idea behind the CWT is to apply the wavelet as a band pass filter to the time series. The wavelet is stretched in time by varying its scale, s, so that η = st, and normalizing it to have unit energy. The CWT of a time series, X_n_, n = 1,2,…,N with uniform time step δt, is defined as the convolution of X_n_ with the scaled and normalized wavelet [23,24].

$$W_{n}^{X}(s)=\sqrt{\frac{\delta t}{s}}\sum_{n^{\prime }=1}^{N}X_{n^{\prime }}\psi_{0}\left[\left(n^{\prime }-n\right)\frac{\delta t}{s}\right]$$

Wavelet power [24]:

$${\left|W_{n}^{X}(s)\right|}^{2}$$

The comparison of the CWT of leptospirosis incidence with meteorological parameters demonstrated clearly common features in the wavelet power. In order to check the possibility of common power, the cross-wavelet transform was performed. The Cross Wavelet Transform (XWT) finds regions in time frequency space where the time series show high common power. The cross wavelet transform of two time series X_n_ and Y_n_ is defined as [23,24].

$$W^{XY}=W^{X}W^{Y*},$$

where * denotes complex conjugation.

Cross wavelet power [23,24]: |W^XY^|

In order to check the possibility of having a causality effect, the wavelet coherence (WTC) was calculated. Wavelet coherence is defined as the square of the cross spectrum normalized by the individual power spectra. This gives a quantity between 1 and 0, and measures the cross-correlation between two time series as a function of frequency. If there are regions in time frequency space with large common power that have a consistent phase relationship, it suggests causality between time series [23,24].

Wavelet coherence [23,24]:

$$R_{n}^{2}(s)=\frac{{\left|S\left(s^{-1}W_{n}^{XY}(s)\right)\right|}^{2}}{S\left(s^{-1}{\left|W_{n}^{X}(s)\right|}^{2}\right).S\left(s^{-1}{\left|W_{n}^{Y}(s)\right|}^{2}\right)}$$

Where S is a smoothing operator.

In order to find the leading or lagging time, the time series were reconstructed for the period which gives the maximum power. Because wavelet transform is a band pass filter with a known wavelet function, it is possible to reconstruct the original time series. Reconstructed time series [23,24]:

xn=δjδt12Cδψ0(0)∑j=0Jℜ{Wn(sj)}sj12

Where, *ψ*_0_(0) removes the energy scaling, while sj12 converts the wavelet transform to an energy density. The factor *C*_*δ*_ is a constant for each wavelet function.

Considering the above equation and by summing over a subset of the scales, we constructed a wavelet filtered time series. In our study the periods which gave the highest coherence among the leptospirosis incidence and the meteorological parameters were identified. The wavelet filtered time series for the period of our study were reconstructed and the lagging times were calculated. The wavelet analysis was done by using MATLAB R2013a software of MATLAB Corporation,U.S.A. Further details about wavelet analysis can be found in these references [22–26].

#### 2.4.2 Cross-correlation coefficients

We calculated cross-correlation coefficients between weekly leptospirosis incidence and weather variables to get an idea of the magnitudes of correlations, with the view of comparing the influence of studied temperature parameters on leptospirosis incidence. We employed SPSS Statistics 20 software (IBM Corporation, USA) for that. We calculated cross-correlations for minus 12 to plus 12 weeks. We selected a 12-weeks limit considering the lag period results of our wavelet analysis results.

## 3. Results

There were 1312 notified cases and the average annual LI for Hambantota district for our study period was 22.1/100,000 population. It was lowest in 2017 (7.9/100,000 population) and highest in 2011 (74.2/100,000 population), as a result of the outbreak enduring March- June 2011.

### 3.1 Time series graphs of aggregated changes of LI and meteorological parameters

**Fig 2:**
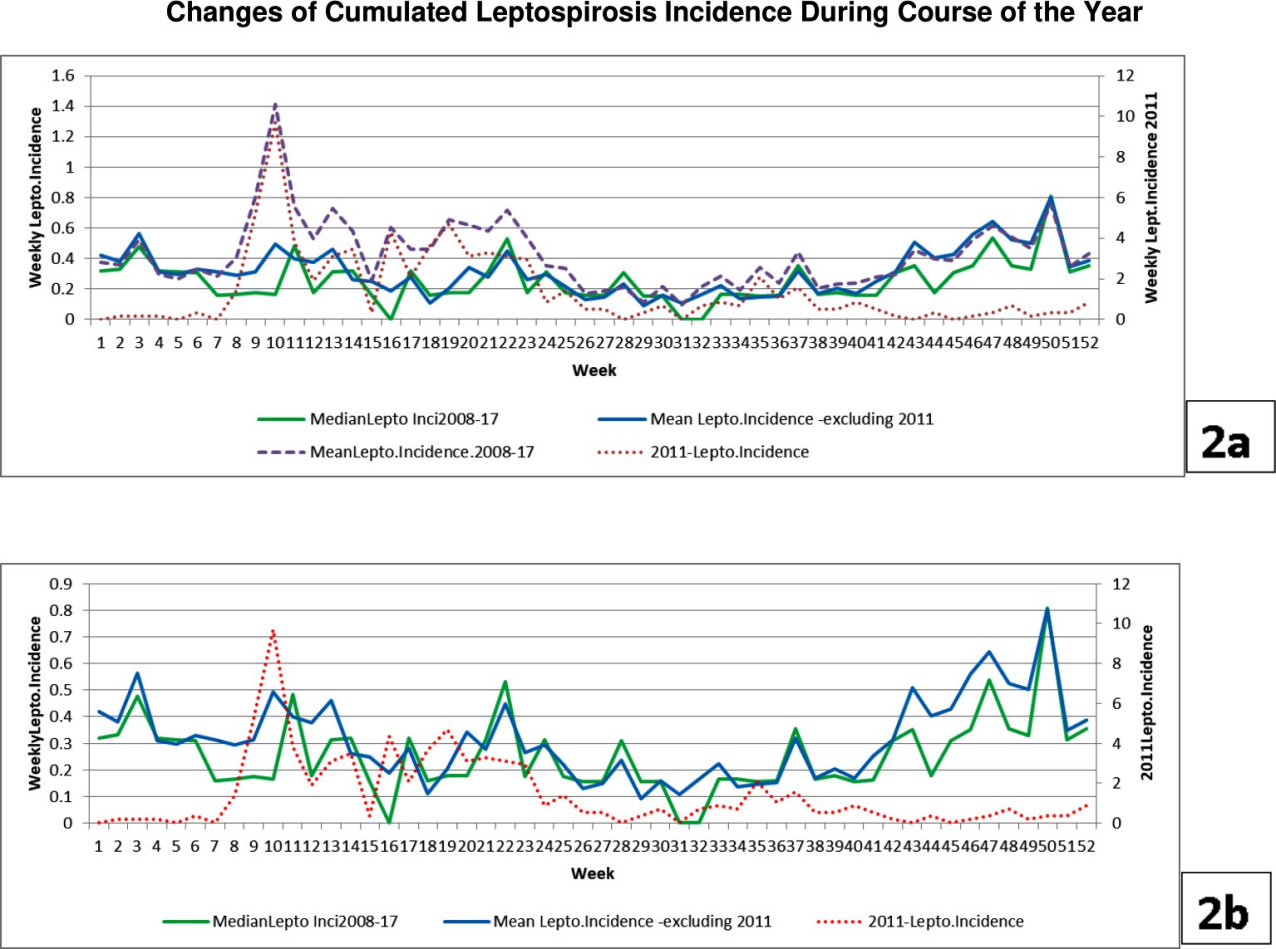
Changes of aggregated weekly leptospirosis incidence (LI) during the course of the year, for 2008–2017. Panel 2a; Changes of aggregated mean (dotted line) and median weekly LI during the course of the year, for 2008–2017 period, mean LI excluding 2011 for the same period and weekly LI during the course of 2011 (all per 100,000 population). x-axis: weeks/ primary y- axis: aggregated LI / LI of 2011 with much high magnitudes depicted in secondary y-axis for clarity: 2b; Changes of aggregated median and mean weekly LI excluding year 2011 during the course of the year, for 2008–2017 period, weekly LI during the course of 2011 (all per 100,000 population).Weekly LI of 2011 with much high magnitudes and a different temporal pattern depicted in secondary y-axis and by a dotted line for clarity.

A time series graph depicting the aggregated changes of LI during the course of 52 weeks of 2011 is shown in Fig 2.

Fig 2A shows that temporal patterns of aggregated mean and median LI are different and temporal patterns of 2011 LI and mean aggregated LI of 2008–2017 are similar. This illustrates how the very high LI of 9-24th week of 2011 influenced the mean aggregated leptospirosis incidence 2008–2017. Fig 2B shows that the median LI 2008–17 and mean LI excluding the year 2011, show similar temporal patterns and magnitudes. The LI of 2011 shown in a different scale shows a very different temporal pattern (and magnitudes). To prevent the influence of the 2011 epidemic distorting the general trends, we appropriated the median leptospirosis incidence for subsequent graphs.

**Fig 3:**
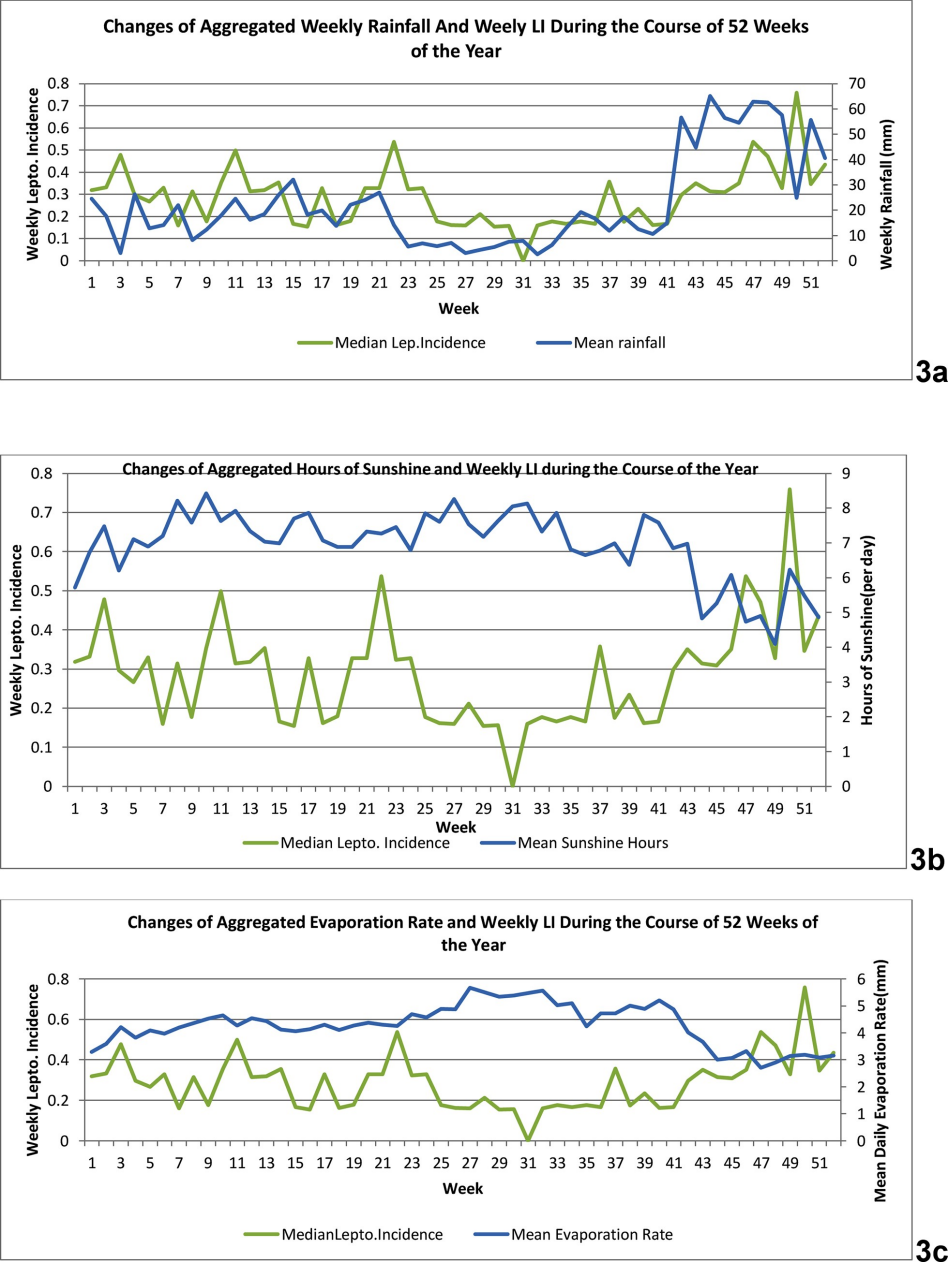
Changes of aggregated weekly rainfall, average hours of sunshine and average evaporation rates versus aggregated median weekly leptospirosis incidence during the course of the year, for 2008–2017. Panel 3a; Changes of aggregated weekly rainfall (in mm) and of average weekly leptospirosis incidence (per 100,000 population) during the course of the year, for 2008–2017. x-axis: weeks/ primary y- axis: median weekly leptospirosis incidence (per 100,000 population). / secondary y-axis: aggregated weekly rainfall (mm). Panel 3b; Changes of aggregated sunshine (hours per day) and of median weekly leptospirosis incidence (per 100,000 population) during the course of the year, for 2008–2017. x-axis: weeks/ primary y- axis: average weekly leptospirosis incidence (per 100,000 population). / secondary y-axis: hours of sunshine (per day). Panel 3c; Changes of aggregated evaporation rate (mm per day) and of median weekly leptospirosis incidence (per 100,000 population) during the course of the year, for 2008–2017. x-axis: weeks/ primary y- axis: median weekly leptospirosis incidence (per 100,000 population). / secondary y-axis: evaporation rate (mm per day).

Fig 3 consists of three panels and illustrates the changes of aggregated weekly rainfall, average hours of sunshine and average evaporation rates versus aggregated median weekly LI during the course of the year, for 2008–2017.

Panel 3a shows the changes of aggregated weekly rainfall and median of weekly LI during the course of the 52 weeks of the year, for 2008–2017. It depicts how the periods of low rainfall during weeks 23–34 were followed by low LI and periods of high rainfall during weeks 42–52 were followed by a rise of LI after a lag period. Panel 3b depicts how the aggregated mean of daily sunshine hours and median of weekly LI change during the course of the 52 weeks of the year for our period of study. Low sunshine hours after the 43^rd^ week were followed by an increase of LI. Panel 3c elucidates how the aggregated mean of daily evaporation rates and median of weekly LI change during the course of the 52 weeks of the year for the same period. After the evaporation rate rises in weeks 23–40, with a lag period, there is a decline of LI. After the 45^th^ week there is a drop in evaporation rates and a rise of LI.

**Fig 4:**
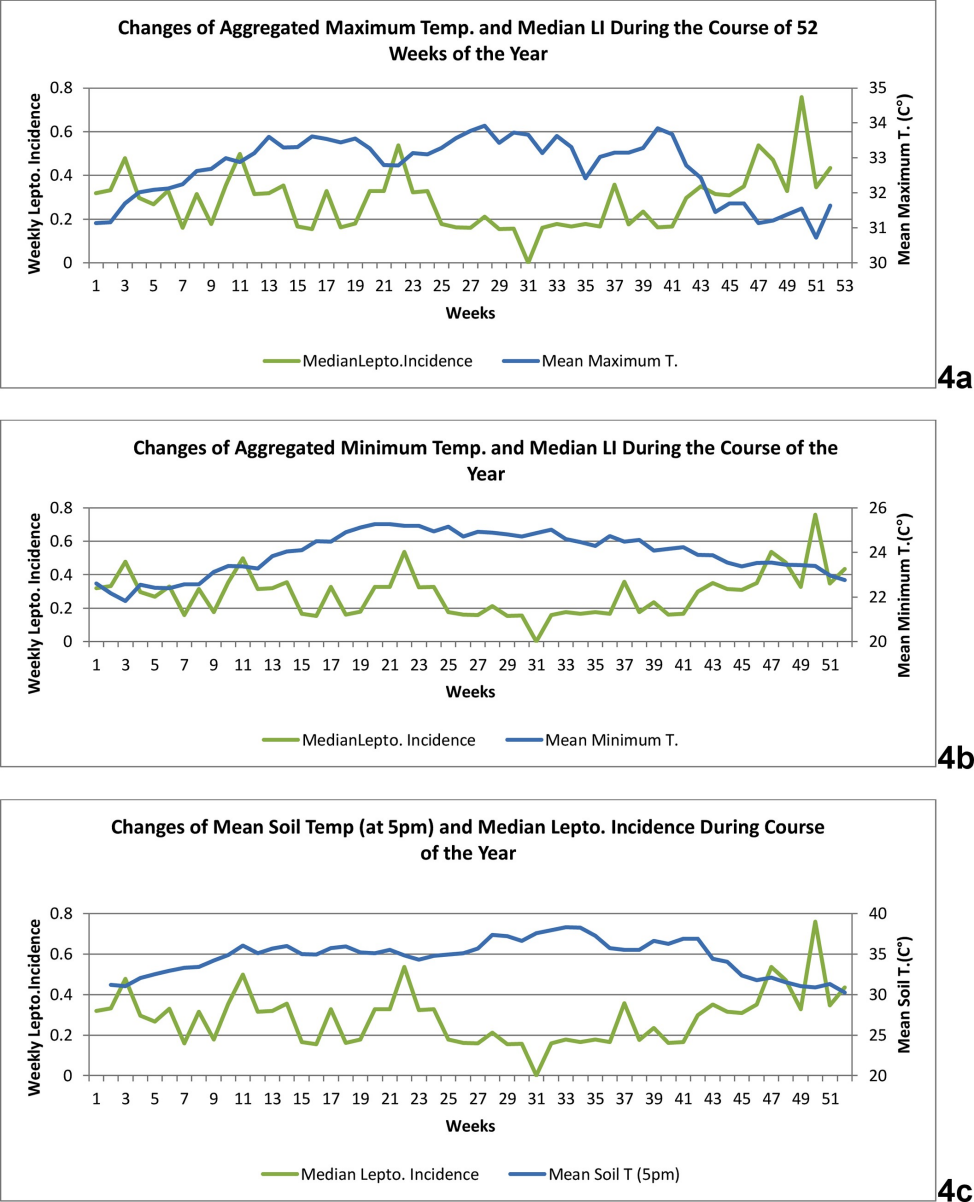
Changes of aggregated weekly minimum and maximum atmospheric temperatures, and soil temperature (at 5 p.m.) versus aggregated median weekly leptospirosis incidence during the course of the year, for 2008–2017. Panel 4a; Changes of aggregated maximum temperature (in degrees Celsius) and of median weekly leptospirosis incidence (per 100,000 population) during the course of the year, for 2008–2017. x-axis: weeks/ primary y- axis: median weekly leptospirosis incidence (per 100,000 population). / secondary y-axis: maximum temperature (in degrees Celsius). Panel 4b; Changes of aggregated mean minimum temperature (in degrees Celsius) and of median weekly leptospirosis incidence (per 100,000 population) during the course of the year, for 2008–2017. x-axis: weeks/ primary y- axis: median weekly leptospirosis incidence (per 100,000 population). / secondary y-axis: minimum temperature (in degrees Celsius). Panel 4c; Changes of aggregated soil temperature (in degrees Celsius) and of median weekly leptospirosis incidence (per 100,000 population) during the course of the year, for 2008–2017. x-axis: weeks/ primary y- axis: median weekly leptospirosis incidence (per 100,000 population). / secondary y-axis: soil temperature (in degrees Celsius).

Fig 4 consists of three panels showing time series graphs of three temperature parameters.

Fig 4A shows the changes of aggregated mean weekly maximum temperature (in degrees Celsius) and of median weekly LI (per 100,000 population) during the course of the year for 2008–2017. During weeks 11–21 and 24–42, the maximum temperature remains high and LI is relatively low. After the 45^th^ week, the maximum temperature declines and that continues until the 3^rd^ week of the next year and then gradually rises. LI is high during this period. During weeks 11–21, despite the high maximum temperature, LI does not decline much. Fig 4B depicts the changes of aggregated weekly minimum temperature (in degrees Celsius) and of median weekly LI during our period of study. As in the graph 4b, when the minimum temperature is high during midyear, the LI is low. The minimum temperature is low at the end of the year, which continues to the first eight weeks of the next year, and LI is high. In spite of the rising minimum temperature during weeks 11–24, there are peaks of LI. Fig 4C portrays the changes of aggregated mean evening soil temperature (in degrees Celsius) and of median weekly LI. The temporal change patterns are similar to that of Fig 2B. When soil temperature rises >35 degrees Celsius LI declines.

**Fig 5:**
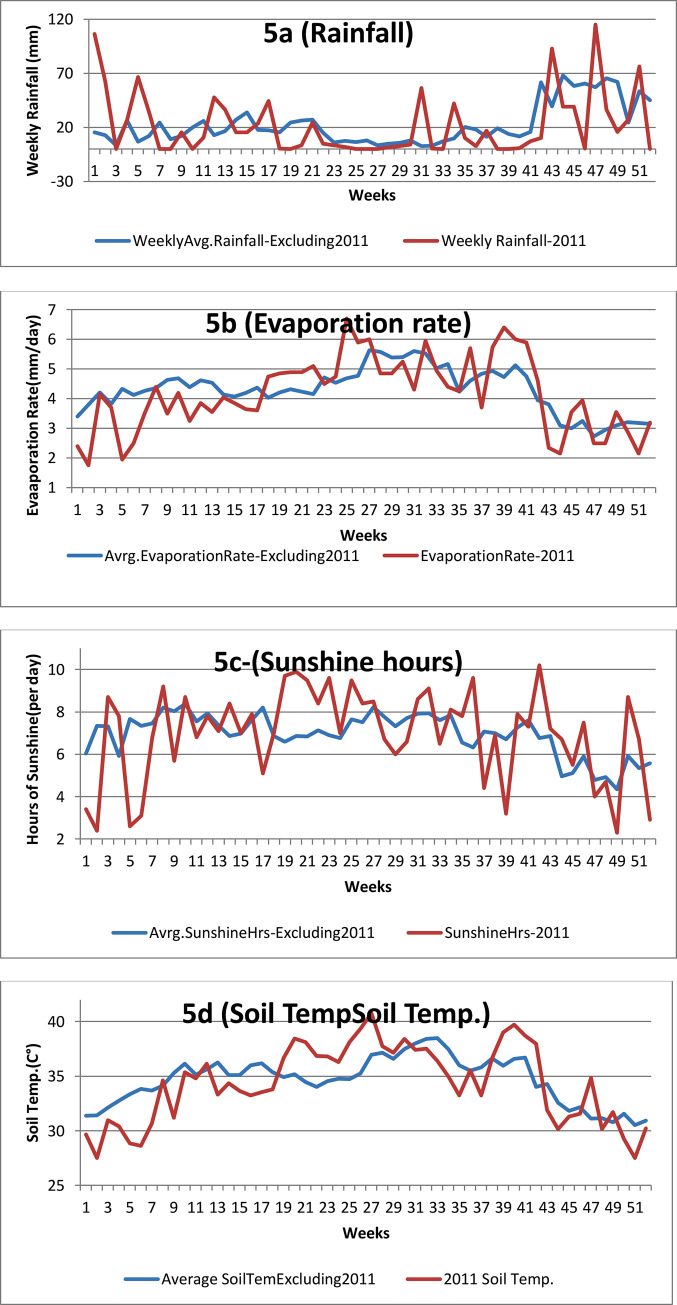
Comparison of weekly weather parameters of 2011 against aggregated averages of those weather parameters of other nine years are illustrated in six panels (a-f) of this figure. Time series of 2011 are shown in maroon whilst aggregated averages of other years are in blue. 5a: Rainfall, 5b: Evaporation rate, 5c: Hours of sunshine, 5d: Soil temperature, 5e: Maximum air temperature, 5f: Minimum air temperature.

Time series graphs of aggregated weather parameters of other nine years against 2011 are illustrated in Fig 5.

According to Fig 5, generally there was lower than average evaporation rates, hours of sunshine, soil and air temperatures and higher than average rainfall during the first 18 weeks of 2011.

### 3.2 Results of wavelet analysis

**Fig 6:**
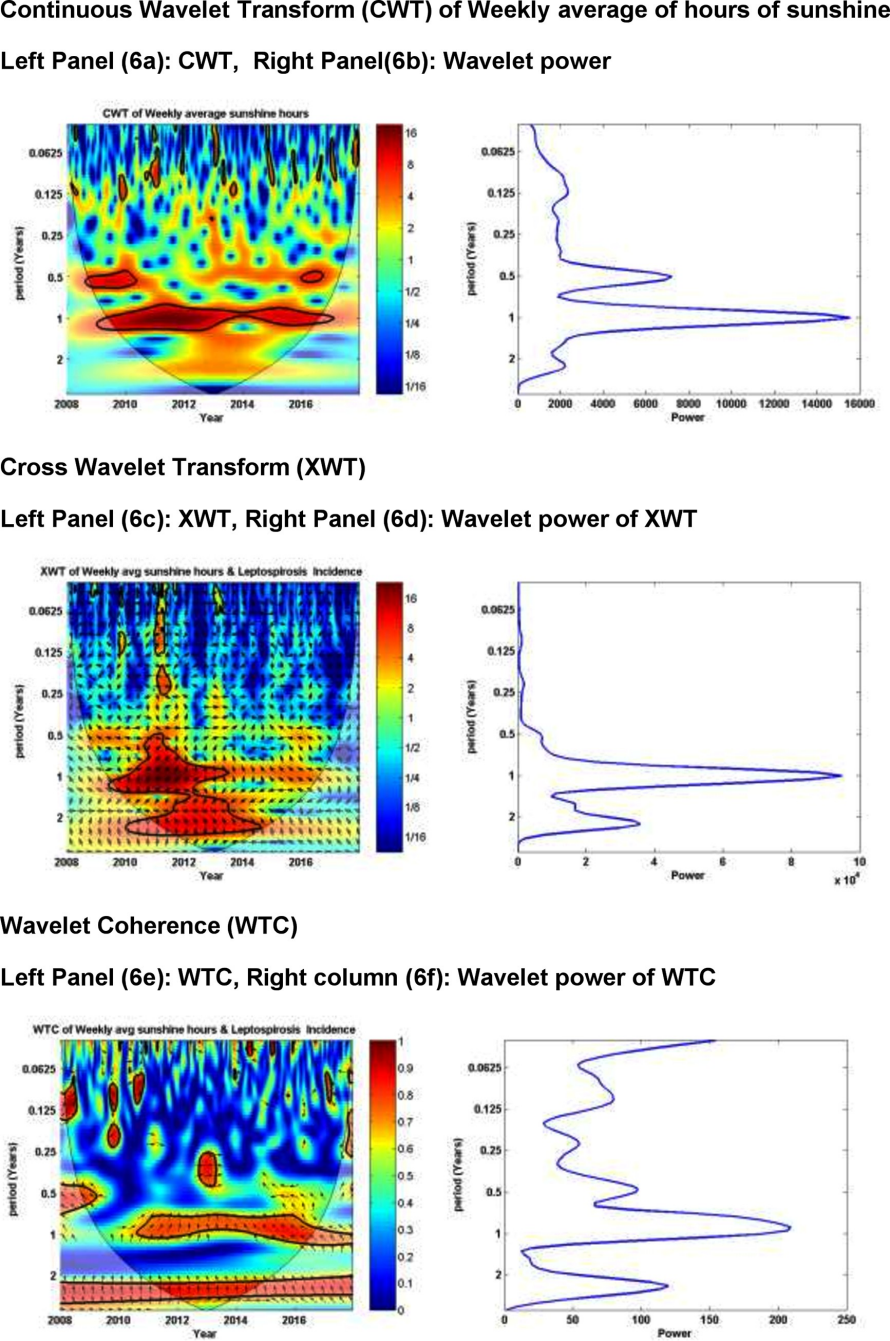
Results of wavelet analyses of weekly average soil temperature (at 5pm) versus weekly leptospirosis incidence for 2008–2017: (Panel 6a) continuous wavelet transform (CWT) variations; (Panel 6b) wavelet power of CWT; (Panel 6c) crosswavelet transform (XWT) variations; (Panel 6d) wavelet power of XWT; (Panel 6e) wavelet coherence (WTC); (Panel 6f) wavelet power of WTC; and (Panel 6g) reconstructed time series for 2006–2015 period.

Fig 6 illustrates the results of wavelet analysis.

The explanation of the Fig 6 is very similar to explanation of wavelet analysis results of two related papers by us [6,23].

Panel 6a of the Fig 6 shows the continuous wavelet transform of the weekly average of hours of sunshine (per day), which expands the time series into time frequency space, whilst panel 6b summarizes the power for each period. Panel 6c shows the cross wavelet transform of weekly minimum temperature with weekly leptospirosis incidence, whereas panel 6d depicts the power for each period. As panel 6e and 6f show, wavelet coherence is greatest between weekly leptospirosis incidence and hours of sunshine for yearly (period) cycles. Color-coded panels on the right side of panels 6a, 6c and 6e illustrate the magnitudes of CWT, XWT, and WTC, in which dark blue and dark red indicate the lowest and highest respectively. The thin U-shaped black lines in 6a, 6c and 6e are the cone of influence. The thick black lines in panels 6a, 6c and 6e are the 5% significance level using the red noise signal model. The arrows in panels 6c and 6e are vectors indicating the phase difference. A horizontal arrow pointing from left to right signifies in the phase and an arrow pointing vertically upward means the second series lags behind the first by 90^0^.

Panel 6g is the reconstructed time series for the period of study (2008–2017). The mean time period between peaks of sunshine hours and subsequent peaks in the weekly leptospirosis incidence in this time series was 11 weeks and that is the average lag period.

### 3.3 Combined summary of wavelet analysis and cross-correlation analysis (SPSS) results

Table 1 gives the combined summary of wavelet analysis and cross-correlation analysis results.

**Table 1:** Combined summary of wavelet analysis and cross-correlation analysis (SPSS) results.

Meteorological Parameter	Results of Wavelet Analysis		Results of Cross-correlation Analysis (SPSS)		
	Average lag and range within brackets	Correlation with Leptospirosis Incidence	Correlation with Leptospirosis Incidence	Highest Cross-correlation Coefficient (lag period within brackets)	
Rainfall	11weeks *(4–22)*	Peaks of leptospirosis incidence occur after peaks of rainfall.	No significant correlation		
Evaporation rate	11weeks *(4–16)*	Peaks of leptospirosis incidence occur after troughs of evaporation rate.	A Negative correlation	-0.201 (5 weeks)	
Maximum temperature	11weeks *(2–18)*	Peaks of leptospirosis incidence occur after troughs of maximum temperature.	A Negative correlation	-0.223 (9weeks)	
Minimum temperature	10weeks *(4–18)*	Peaks of leptospirosis incidence occur after troughs of minimum temperature.	A Negative correlation	-0.193 (8weeks)	
Mean temperature	11weeks *(3–18)*	Peaks of leptospirosis incidence occur after troughs of mean temperature.	A Negative correlation	-0.234 (9weeks)	
Soil temperature	10weeks *(1–19)*	Peaks of leptospirosis incidence occur after troughs of soil temperature.	A Negative correlation	-0.182 (5weeks)	
Hours of sunshine	11weeks *(6–18)*	Peaks of leptospirosis incidence occur after troughs of weekly hours of sunshine.	A Negative correlation	-0.161 (10weeks)	
Monthly Nino 3.4 SST anomaly index	No clear correlation pattern				
Monthly Nino 4 SST anomaly index	No clear correlation pattern				
Monthly DMI anomaly index Correlation 1	2.9 months (0–6)	Peaks of leptospirosis incidence occur after peaks of DMI			
Monthly DMI anomaly index Correlation 2	4 months (1–7)	Peaks of leptospirosis incidence occur after troughs of DMI			

## 4. Discussion

All meteorological parameters studied and DMI were correlated with LI of Hambantota. Different lag periods in our wavelet and SPSS results can be attributed to differences in the analytic technique and an example of the influence of results by analysis technique in similar studies [6,23].

### 4.1 Discussion of time series graph patterns

Annual seasonal change of LI in Hambantota is illustrated in Fig 2. Such seasonality was demonstrated by studies done in other areas of Sri Lanka [6,7]. Graphs like Fig 2 give a good basic idea of changes of LI with annual cycles of the local weather. Our Fig 2 led us to understand how strongly the 2011 outbreak (9-24^th^ week) influenced aggregated mean weekly leptospirosis incidence in this 10 year study. It is an example of how one outbreak with a duration of several weeks happening in unusual months can distort the general temporal pattern and magnitudes of an aggregated incidence graph even in a decade-long study. Many preventive health authorities known to the authors study graphs of aggregated mean monthly/weekly incidence of the past several years and decide the most appropriate month/s of the year to make extra effort to control climate-sensitive infectious diseases like leptospirosis, influenza and dengue. Had we considered only the means of weekly LI for plotting the graph for Fig 2, we would have ended up with a graph with a misleading pattern. We present that as an example of the benefit of looking for both aggregated mean and median to get a more realistic picture in similar studies.

The results of both wavelet analysis and cross-correlation analysis are in general agreement with our time series graphs of correlation between aggregated weekly meteorological parameters and median of weekly leptospirosis incidences during the course of the 52 weeks of the year, for the period of 2008–2017. That elevates the fidelity of our results. Nonetheless, there was no cross-correlation between rainfall and LI (Table 1). We give more emphasis to our wavelet analysis results (more suitable to detect such non-linear and non-stationary correlations) and conclude that peaks of rainfall are followed by peaks of LI. Our aggregated time series graph (Fig 3) also supports this conclusion.

In our aggregated time series graphs (Fig 4), during weeks 11–21 the maximum temperatures and soil temperature remain high, during 15–21 the minimum temperatures is high. Yet after a pause there is a peak of LI. This appears to contradict our wavelet results. However, we think there is a plausible explanation. The harvesting of rice in the main (Maha) season of the year happens early in this period. During the harvesting season cultivators and many others who usually engage in other occupations also work in rice paddies, exposing themselves to leptospires, and Hambantota receives some rain from evening thunderstorms of the first inter-monsoon season. The net effect of these can lead to the rise of LI.

### 4.2 Comparison of results of the present study with results of past studies from Sri Lanka and elsewhere

The results of the present study are generally in agreement with past studies done in Sri Lanka and several other countries except for temperature parameters and for sunshine hours [4,6–8]. We discuss the differences of results regarding temperature parameters and for hours of sunshine in details in 4.4 below.

Among factors that affect leptospires’ survival in soil; pH, salinity, texture and the microbiota of soil all are also influenced by local weather [12,27–29]. The persistence of leptospires in water bodies is influenced by pH, solutes in water [29]. The composition of fauna and common serovars of leptospires differs in different climatic and ecological regions in Sri Lanka [12]. The duration and intensity of urinary shedding of leptospires varies between mammals from species to species [30]. Even in the same rodent species it varies markedly for different serovars of leptospires [31]. Leptospirosis species and serovars responsible for outbreaks in the wet zone (Kandy) and the dry zone (Hambantota) of Sri Lanka are different [32]. Longevity in the environment varies among different pathogenic species of leptospires and longevity affects lag periods [27]. The differences in lag periods compared to past studies including our past study done in Kandy may be partially explained by differences of influence by the factors discussed above. Nonetheless, Kandy city is a mere 142km from Hambantota town as the crow flies in the same country. Behaviors and practices that put people at risk of contracting leptospirosis, protective measures they take, healthcare seeking and accessibility and preventive health mechanisms of Hambantota and Kandy are more similar than those between populations of two nations. Hence comparing the effect of weather on LI in those two populations using the same source of data and methodology is interesting. Soil in dry zone coastal plains like Hambantota generally has higher pH and salinity than Kandy in the wet zone highlands [33]. The land use patterns of the two districts differ. Kandy gets more rain, more frequently [6]. Thus, the norm is soil remains more moist than in Hambantota. In Kandy we saw troughs of weekly rainfall followed by troughs of LI but no peaks after peak correlation [6]. In Hambantota, with normally dry soil, we see peaks of leptospirosis incidences following peaks of rainfall, but no troughs after trough correlation. Nonetheless following weeks with many wet days and days with rainfall >100mm, there were peaks of LI in Kandy [6]. A recent report from neighboring India demonstrated a positive correlation with concentration of leptospires in rice paddies and rainfall [28]. Moreover, concentration of Pseudomonas bacteria in rice paddies that shows antagonistic activity towards leptospires *in vitro* has shown negative correlation with rainfall [28]. These findings support our results.

During the 2008 epidemic that affected mostly the wet zone districts, including Kandy, Hambantota was not severely affected [7,32].

### 4.3 Discussion of the 2011 outbreak

Heavy rains and resulting floods affected Hambantota and several other districts of Sri Lanka (mostly in the dry zone) in January 2011 [32,34]. Hambantota received a heavy rainfall during 13–18 weeks of 2011 as well. As Fig 5 illustrates, other meteorological parameters studied were also conducive for leptospirosis transmission during the first 18 weeks of 2011. There were lower than average soil and air temperatures, hours of sunshine and evaporation rates (that were shown to be favorable for leptospirosis transmission locally). We believe this combination has contributed to the outbreak from the 9-24^th^ week of 2011 (depicted by Fig 2B). However, only a smaller spike of incidence resulted in Kandy in early 2011 [6]. In Kandy a few dams across the main river prevented major floods, and due to hill terrain the overflow of minor waterways quickly subsided but Kandy had more landslides in 2011 [34]. Deep soil and rocks dispersed after landslides do not contain leptospires. Factor/s other than weather present in Hambantota but not in Kandy may have contributed to Hambantota outbreak as well.

Leptospirosis outbreaks subsequent to deluges were reported worldwide [1–4,6,8,11,13]. Flood water disperses leptospires (and drowned infected animals) so more people come into contact with them, as local people do not wear boots when walking through flood water or on paths that were recently flooded, and do not wear gloves when cleaning the residual debris following floods. Walking through flood water increased risk leptospirosis fifteen fold in one case-control study [11]. Flood-displaced people often live in poor sanitary conditions. During heavy rains many local people herd their livestock from fields to their backyard and dogs that usually stay outdoors tend to stay indoors, increasing the risk of contracting leptospirosis. When burrows of peri-domestic rodents get inundated, they also tend to come closer to people. Heavy rains and the inundation of rice paddies occurred closer to ‘Maha’ harvesting season in 2011. Closer to harvesting season, rodents living near rice paddies are known to move paddies, so those were more likely to have been contaminated with their urine [18,35]. After harvesting, children play and cattle graze in local rice paddies sometimes. Those factors also may also have contributed to the 2011 outbreak. According to the Fig 5, during the last 10 weeks of 2011 the general weather of Hambantota was also conducive for leptospirosis transmission yet there was no outbreak. There were floods in many districts of the country including Hambantota in May 2017, but there was no major leptospirosis outbreak in Hambantota subsequently. As discussed later in 4.10, leptospirosis incidence is the net effect of weather and many other factors. If other factors also simultaneously significantly contribute to spread of leptospirosis, a major outbreak ensues in favorable weather conditions, as occurred in early 2011. It is interesting to note the similarity of this phenomenon with the classic Swiss cheese model for human errors [36]. We did not find any evidence of marked change of most the above mentioned non-weather factors of Hambantota within 2010–12. We suspected a possible outbreak among animals during the same time may have released more than the usual amount of leptospires or unusual species/ serovar to the soil contributing to early 2011 outbreak, and looked for evidence. Searching through volumes 3–4 of the Veterinary Epidemiological Bulletin of Sri Lanka and a few visits to Sri Lanka Department of Animal Production and Health failed to find any record of a leptospirosis outbreak among domestic animals in Hambantota (and the Kurunegala and Anuradhapura districts; we discuss these below) from July 2010- April 2011. Most rural smallholder farmers rarely seek veterinary services thus only a small fraction of diseases get notified. Nonetheless, we found a hint that a change of the commonest leptospira species is responsible. *Leptospira interrogans* has been the commonest species responsible for human cases and not a single case of *L*. *kirschneri* was reported among the people of Sri Lanka from 1960s until 2008. Three human cases of *L*. *kirschneri* were detected between 2008–2011, all of which were from the from the Kandy district according to very limited test results available [32,37,38]. Therefore we can presume that only a small percentage of Sri Lankans possess any immunity against serovars of *L*. *kirschneri* that do not overlap with serovars of *L*.*interrogans*. A study conducted in Anuradhapura, another dry zone rice cultivating district, during 2011 post-flood outbreak showed *L*. *kirschneri* was the predominant species responsible and those authors attribute the 2011 outbreak (in the dry zone) of Sri Lanka to *L*. *kirschneri* [32]. An interesting analogue is that the introduction of uncommon sero/geno types of dengue virus to Sri Lanka has also contributed to dengue outbreaks [23,39,40]. In September 2010, kidney samples of 164 cattle, taken from the meat inspection hall of Colombo city, were analyzed in another study. Those cattle were inspected and approved as disease free by the Municipal Veterinarian Service. None of them originated from Hambantota. Kidneys from 20 were found to harbor pathogenic Leptospira species. Out of 20, seven had *L*. *kirshneri* and all seven cattle originated from Anuradhapura and Kurunegala districts in the dry zone of Sri Lanka [41]. We revisited weekly epidemiology reports from which we obtained patient data and found that Kurunegala reported the highest count of leptospirosis cases of the country during the same (2011) post-flood period. Although no information is available of any identification of responsible species in human or animal leptospirosis cases from Hambantota during 2010–2011, and Anuradhapura and Kurunegala districts are not close to Hambantota, we think the information available can lead us to a plausible hypothesis. This is that is a *L*. *kirschneri* outbreak among cattle (and other animals) may have released an unusually large amount of leptospires into the environment and favorable weather and flooding may have facilitated an outbreak among people in early 2011 in Anuradhapura, Kurunegala and possibly in Hambantota and other districts. We found a similar scenario in literature [42]. A leptospirosis outbreak in 2004 in Guadeloupe in an unusual season was attributed to hot and wet conditions created by ENSO. Interestingly, a locally uncommon leptospira serogroup becomes common among patients following its increased incidence among mice during this period. In 2000 there were also heavy rains in Guadeloupe. Conversely, that serogroup was reported from a smaller percentage of patients, and there was no outbreak among people. Our study illustrates that when we study correlation patterns of LI with weather, while simultaneously considering local non-weather factors gives a more realistic and useful picture. We have an interesting analogue from a past study. We have demonstrated the combination of an abundance of infective agent (infected hosts) with the vector in the environment and weather conducive for transmission resulting in a major nosocomial dengue outbreak [39].

### 4.4 Discussion of correlation between LI and temperature parameters, hours of sunshine and evaporation rate

Most of the past studies, including our Kandy study, in contrast to our results, report a rise of LI following rise of temperature [6,8]. We have a plausible explanation for this. The average mean and maximum ambient temperatures of Hambantota during our period of study respectively were 28.3 degrees Celsius and 32.7degrees Celsius. The average soil temperature in the evenings was 34.6 degrees Celsius. The optimum growth temperature for leptospires in the laboratory is 28°C—30°C [43]. Thus the further rise of temperatures above the optimum range may have been unfavorable for the survival of leptospires in Hambantota. That explains our results of troughs of LI following peaks of all three of the temperature parameters we studied. Maximum and mean atmospheric temperatures had cross-correlations of the highest magnitudes, indicating a significant detrimental effect of higher than optimum temperatures on survival of leptospires. Although leptospires inhabit soil, the magnitude of cross-correlation was less for soil temperature. We anticipated the opposite. We presume the reason may be that soil temperature in the environment can vary even within a few meters distance depending on vegetation (shade) and other factors, thus variation from the average value is greater compared to ambient temperature from place to place. During hot and dry times, people, domestic animals and wild animals who come to human habitats share dwindling water sources for bathing and drinking, thus increasing the risk of leptospirosis transmission. Our results indicate that this factor plays a lesser role in Hambantota. A study from Thrissur district, Kerala, India with similar high maximum temperatures like Hambantota but also with high rainfall shows that high maximum temperatures have a negative correlation with leptospirosis case numbers [44]. In Kandy, with an average temperature of 25°C, a rise of temperatures means moving towards the optimum range. That explains the peaks of LI following peaks of temperature parameters in Kandy district [6]. A study conducted in Reunion Island, which has a lower average temperature than Kandy, showed positive correlation between LI and average temperature [45]. The same explanation is applicable to them as well.

The Reunion Island study demonstrated a negative correlation between global solar radiation and LI and that is in agreement with our results [45]. However, there was no correlation between sunshine and LI in a study done in Korea [46]. Those authors explain that result in terms of leptospires possessing self-defenses with the capability of recovering from damage by ultraviolet rays of the Sun. The auto-repair capability of leptospires becomes inefficient after >6 hours of exposure to ultraviolet rays [47]. The average daily sunshine in Korea is generally shorter than that of Hambantota. For example, it is 5.7 hours for Seoul in contrast to 6.9 hours in Hambantota [48]. Our results indicate that the effects of prolonged sunshine may be overwhelming leptospires’ auto-repair capability against ultraviolet radiation in Hambantota. High evaporation rates and soil temperatures associated with prolonged sunshine also may have a negative effect on LI. The correlation obtained for evaporation rate was as we expected and we think future researchers may consider including that in similar studies.

### 4.5 Discussion of correlation between ENSO, IOD and LI

Our result of no clear correlation between both ENSO indices and LI was unexpected. Interaction between the coupled ocean atmosphere phenomena and other atmospheric phenomena determine local rainfall [19]. The rainfall of Angunakolapelessa, one of the weather stations of the present study, showed a dual correlation, positive correlation with Nino3.4 SST anomaly in October-December period and a negative correlation in January-March period inconsistently [19]. However, the rainfall of two other locations in the Hambantota district, namely Kirama and Ambalantota were not meaningfully modulated by ENSO, but IOD to a lesser degree affected the rainfall of all three locations according to that study [19]. That agrees with our results. We saw a rise of LI following both troughs and peaks of DMI, an uncommon correlation pattern. A similar result was shown by a past study from Columbia that studied the correlation between ENSO and LI using ONI = Oceanic Nino Index [49]. Seventeen municipalities had a rise in leptospirosis cases during La Nina periods (troughs of Nino 3.4 and ONI). Of those, seven also had an increase of leptospirosis during the El Nino (peaks of Nino 3.4) month as well [49]. The peak of DMI index in January 2011 was followed by the early March peak of LI in our reconstructed time series in wavelet analysis specifying IOD’s contribution to the 2011 outbreak. A past study shows DMI peaks enhancing rainfall during the ‘Maha’ agriculture season of Sri Lanka [20]. The 2011 floods occurred during the latter part of this season. There was a trough in both Nino indices in September 2010 (a La Nina) and even though there was no clear *en block* correlation pattern with Nino indices and LI, this particular trough was followed by the early 2011 peak of LI in our reconstructed time series. Another study shows that the late 2010 La Nina contributed to the 2011 rainfall (and floods) in Hambantota [50]. Hence ENSO may also have contributed to the early 2011 outbreak. The same La Nina was attributed to floods and subsequent rise of leptospirosis in Columbia as well [49].

### 4.6 Influence of wild animals on LI in Hambantota and Sri Lanka

A study from Sarawak, Malaysia shows that people with frequent contact with wild animals show higher seropositivity for leptospirosis [1]. The borders of three major national parks extend to Hambantota and wild animals frequently come into contact with people and livestock, especially during dry seasons. That may have contributed to the high LI of Hambantota.

Sri Lanka is home to 98 terrestrial mammal species and 33 of them are rodents or shrews [51]. Reptiles and birds can also host leptospires [52]. No information is available about non-mammal vertebrates’ contribution to leptospires in soil and water in human habitat in Sri Lanka. We only have a very limited understanding of contribution by a few mammal species in Sri Lanka [12,38].

### 4.7 Other information needed to forecast future major outbreaks

Although we detected Leptospirosis incidences correlations with all the weather parameters studied, in order to forecast future major outbreaks (especially magnitudes of peak incidences) the inclusion of other parameters like changes of common species/serovars in circulation and leptospire concentration changes in environment in outbreak prediction models will be useful [42]. Some past models based solely on weather parameters were not good enough to predict all large peaks of LI [45]. Those peaks have public health significance, as our results demonstrated. The generation of evidence by facilitating the diagnosis of serovars /species of all possible cases among people and animals at least at important sentinel sites, and serial soil and water sampling will be helpful to generate the lacking data, but need considerable resources to implement. Monitoring outbreaks of leptospirosis among people and animals under one health concept may be helpful to forecast risk of outbreaks among people [53].

### 4.8 How climate change would influence LI of Hambantota

Droughts and flash floods are associated with ongoing global climate changes and deforestation. Sri Lanka has experienced these more frequently in the recent past [54]. Hambantota is the district with highest exposure to climate change in Sri Lanka, according to one analysis [54]. Flash floods may result in more leptospirosis outbreaks as in 2011 in Hambantota. Nonetheless, the rise of the count of days with maximum temperature > 34^0^ C in Hambantota due to climate change is going to be unfavorable for leptospirosis transmission [54].

### 4.9 Relevance of our findings to leptospirosis preventive work

The reported LI of Hambantota estimated by us (22.1/100,000) using data from the same source is far above the national annual reported case incidence of 5.4/100,000 stated by Sri Lanka Ministry of Health [3]. Nevertheless, notified cases are a fraction of the true incidence rate and incidence of Sri Lanka was reported to be among the highest in the world [9]. Therefore, more attention should be given to augment existing preventive work in Hambantota, especially when the weather is conducive for leptospirosis transmission and when people engage in high risk activities like rice planting. According to a systematic review article, chemoprophylaxis with doxycycline for high risk populations can effectively prevent leptospirosis incidence among them with minimal adverse effects and is recommended by the Health Ministry of Sri Lanka as well [3,55]. A study conducted in the nearby Galle district illustrated that less than one third of the farmers surveyed knew the correct recommendations and used doxycycline chemoprophylaxis [56]. We recommend the vigorous promotion of the use of prophylactic doxycycline among vulnerable people such as rice cultivators using mass media, especially when the weather is conducive for leptospirosis transmission (i.e;October–January in ‘Maha’ season) and during heavy rains. Such methods can be adopted for leptospirosis preventive work in other parts of Sri Lanka and elsewhere as well, with some local modifications. Homoeoprophylaxis with an oral vaccine proved to be effective in controlling leptospirosis, especially the peak incidence following heavy rain in another tropical island nation Cuba, but did not gain wide acceptance [57]. The availability of an effective oral vaccine giving rise to a long lasting immunity will be useful to blunt outbreaks like the 2011 outbreak and thus will be useful to susceptible people worldwide. It is very important because leptospirosis is estimated to result in more deaths than other hemorrhagic fevers and a large fraction of those affected are in the prime age of their working life [5]. Hence this deserves the attention of the preventive health community. The use of personal protection such as gloves and boots are considered as an alien practice by most Sri Lankan farm and other manual workers, especially by the poor in rural areas like Hambantota, to the best knowledge of the authors. During the ongoing COVID-19 pandemic (2020) local authorities managed to promote wearing face masks in public, another practice alien to local culture, by the combination of energetic and sustained mass education and enforcement campaigns to become a norm. COVID-19 phobia created by the news media also helped. That indicates that similar methods have the potential to be used for promoting personal protection against leptospirosis as well.

### 4.10 Limitations of the present study

The net sum of contributions of meteorological factors and independent variables, such as herd immunity and the socio-economic status of the population, fluctuations of abundance of infected natural hosts (especially rodents) in the area, pattern of land use, garbage disposal methods, the percentage of the population engaged in high risk activities occupationally and for leisure, the population’s practices regarding personal protection against leptospirosis, the introduction of new species/serovars of pathogen into the population, the percentage of population with symptoms who seek hospital care and get reported and outbreaks among animals of the area and efficacy of preventive programs contribute to the leptospirosis incidence of an area [1–4,6,8,13,27]. In this present study we considered details of only the meteorological factors. Nevertheless, many of those non-meteorological factors are indirectly modulated by the weather and some of them do not change considerably on a weekly/monthly basis.

Notified cases here are hospitalized in-patients. To the best knowledge of authors, some hospitalized cases are not notified. As the majority of the cases are not serologically/PCR test confirmed, there may be some misdiagnosed cases entered into the data as well [6]. Nevertheless, notified cases are the best practical option for a long-term retrospective study in Sri Lanka.

## 5. Conclusions

LI of Hambantota is well above national incidence. All seven weather parameters studied and the index of IOD (DMI) were correlated to LI of Hambantota. Favorable weather contributed to the 2011 leptospirosis outbreak. This may be the first long-term study demonstrating the soil temperature, evaporation rate and IOD are correlated with LI. When studying the correlation between weather and LI, giving attention to relevant non-weather factors also helps to get a better understanding. The correlation pattern of LI with temperature parameters differs from many similar studies in the past and the reason may be that the temperatures in Hambantota are often above the optimum temperature range for leptospires’ survival. We recommend enhancing leptospirosis control programs in Hambantota.

## References

[1] Soo ZM, Khan NA, Siddiqui R. Leptospirosis: Increasing importance in developing countries. Acta tropica. 2020 1 1;201:105183 https://pubmed.ncbi.nlm.nih.gov/31542372/. 10.1016/j.actatropica.2019.105183 31542372

[2] World Health Organization. Human leptospirosis: guidance for diagnosis, surveillance and control. World Health Organization; 2003 https://apps.who.int/iris/bitstream/handle/10665/42667/WHO_CDS_CSR_EPH_2002.23.pdf.

[3] Epidemiology Unit.Ministry of Health- Sri Lanka. National Guidelines on Management of Leptospirosis. Ministry of Health, Nutrition and Indigenous Medicine-Sri Lanka;2016.

[4] Mwachui MA, Crump L, Hartskeerl R, Zinsstag J, Hattendorf J. Environmental and behavioural determinants of leptospirosis transmission: a systematic review. PLoS Negl Trop Dis. 2015 Sep 17;9(9):e0003843 https://www.ncbi.nlm.nih.gov/pmc/articles/PMC4574979/. 10.1371/journal.pntd.0003843 26379035 PMC4574979

[5] Costa F, Hagan JE, Calcagno J, Kane M, Torgerson P, Martinez-Silveira MS, et al Global morbidity and mortality of leptospirosis: a systematic review. Plos negl trop dis. 2015 9 17;9(9):e0003898 https://www.ncbi.nlm.nih.gov/pmc/articles/PMC4574773/. 10.1371/journal.pntd.0003898 26379143 PMC4574773

[6] Ehelepola ND, Ariyaratne K, Dissanayake WP. The correlation between local weather and leptospirosis incidence in Kandy district, Sri Lanka from 2006 to 2015. Global health action. 2019 1 1;12(1):1553283 https://www.ncbi.nlm.nih.gov/pmc/articles/PMC6327921/. 10.1080/16549716.2018.1553283 31154987 PMC6327921

[7] Robertson C, Nelson TA, Stephen C. Spatial epidemiology of suspected clinical leptospirosis in Sri Lanka. Epidemiology & Infection. 2012 Apr;140(4):731–43. https://pubmed.ncbi.nlm.nih.gov/21676347/. 10.1017/S0950268811001014 21676347

[8] Dhewantara PW, Lau CL, Allan KJ, Hu W, Zhang W, Mamun AA, et al Spatial epidemiological approaches to inform leptospirosis surveillance and control: A systematic review and critical appraisal of methods. Zoonoses and public health. 2019 3;66(2):185–206. https://europepmc.org/article/med/30593736?testing&client = bot. 10.1111/zph.12549 30593736

[9] Warnasekara J.N. and Agampodi S.B. Leptospirosis in Sri Lanka. Sri Lankan Journal of Infectious Diseases. 2017 Vol.7 (2):67–75. 10.4038/sljid.v7i2.8155.

[10] Levett P. N. Leptospirosis. Clinical microbiology reviews.2001 14(2), 296–326. 10.1128/CMR.14.2.296-326.2001 11292640 PMC88975

[11] Gubler DJ, Reiter P, Ebi KL, Yap W, Nasci R, Patz JA. Climate variability and change in the United States: potential impacts on vector-and rodent-borne diseases. Environmental health perspectives. 2001 5;109(suppl 2):223–33. https://pubmed.ncbi.nlm.nih.gov/11359689/. 10.1289/ehp.109-1240669 11359689 PMC1240669

[12] Gamage CD, Sato Y, Kimura R, Yamashiro T, Toma C. Understanding leptospirosis eco-epidemiology by environmental DNA metabarcoding of irrigation water from two agro-ecological regions of Sri Lanka. PLOS Neglected Tropical Diseases. 2020 7 23;14(7):e0008437 https://pubmed.ncbi.nlm.nih.gov/32701971/. 10.1371/journal.pntd.0008437 32701971 PMC7377381

[13] Goarant, C., Trueba, G., Bierque, E., Thibeaux, R., Davis, B. and De la Peña Moctezuma. Leptospira and Leptospirosis. In: J.B. Rose and B. Jiménez-Cisneros, (eds) Global Water Pathogen Project. http://www.waterpathogens.org (A. Pruden, N. Ashbolt and J. Miller (eds) Part 3 Bacteria) 2019. http://www.waterpathogens.org/book/leptospira-and-leptospriosis Michigan State University, E. Lansing, MI, UNESCO. 10.14321/waterpathogens.26.

[14] Fall MW, Fiedler LA. Rodent control in practice: tropical field crops Animal and Plant Health Inspection Service. USA Department of Agriculture 2015 https://pdfs.semanticscholar.org/1ad8/673a7aa78d7a3f66d917c0fd8d3a99ae5b21.pdf.

[15] Benacer D, Zain SN, Sim SZ, Khalid MK, Galloway RL, Souris M, et al Determination of Leptospira borgpetersenii serovar Javanica and Leptospira interrogans serovar Bataviae as the persistent Leptospira serovars circulating in the urban rat populations in Peninsular Malaysia. Parasites & vectors. 2016 12 1;9(1):117 https://pubmed.ncbi.nlm.nih.gov/26927873/. 10.1186/s13071-016-1400-1 26927873 PMC4772511

[16] Orrock JL, Danielson BJ. Temperature and cloud cover, but not predator urine, affect winter foraging of mice. Ethology. 2009 7;115(7):641–8.

[17] Schønning MH, Phelps MD, Warnasekara J, Agampodi SB, Furu P. A Case–Control Study of Environmental and Occupational Risks of Leptospirosis in Sri Lanka. EcoHealth. 2019 9 1;16(3):534–43. https://pubmed.ncbi.nlm.nih.gov/31664587/. 10.1007/s10393-019-01448-w 31664587

[18] Bambaradeniya CN, Edirisinghe JP, De Silva DN, Gunatilleke CV, Ranawana KB, Wijekoon S. Biodiversity associated with an irrigated rice agro-ecosystem in Sri Lanka. Biodiversity & Conservation. 2004 Aug 1;13(9):1715–53.

[19] Burt TP, Weerasinghe KD. Rainfall distributions in Sri Lanka in time and space: an analysis based on daily rainfall data. Climate. 2014 12;2(4):242–63. 10.3390/cli2040242.

[20] Zubair L, Rao SA, Yamagata T. Modulation of Sri Lankan maha rainfall by the Indian Ocean dipole. Geophysical Research Letters. 2003 1;30(2). http://water.columbia.edu/files/2011/11/Zubair2003Modulation.pdf.

[21] Zubair L, Siriwardhana M, Chandimala J, Yahiya Z. Predictability of Sri Lankan rainfall based on ENSO. International Journal of Climatology: A Journal of the Royal Meteorological Society. 2008 1;28(1):91–101. 10.1002/joc.1514

[22] Cazelles B, Chavez M, Berteaux D, Ménard F, Vik JO, Jenouvrier S, Stenseth NC. Wavelet analysis of ecological time series. Oecologia. 2008 5 1;156(2):287–304. https://pubmed.ncbi.nlm.nih.gov/18322705/. 10.1007/s00442-008-0993-2 18322705

[23] Ehelepola ND, Ariyaratne K, Buddhadasa WM, Ratnayake S, Wickramasinghe M. A study of the correlation between dengue and weather in Kandy City, Sri Lanka (2003–2012) and lessons learned. Infectious diseases of poverty. 2015 12 1;4(1):42 https://www.ncbi.nlm.nih.gov/pmc/articles/PMC4581090/. 26403283 10.1186/s40249-015-0075-8 PMC4581090

[24] Grinsted A, Moore JC, Jevrejeva S. Application of the cross wavelet transform and wavelet coherence to geophysical time series. https://core.ac.uk/download/pdf/52753743.pdf.

[25] Jevrejeva S, Moore JC, Grinsted A. Influence of the Arctic Oscillation and El Niño‐Southern Oscillation (ENSO) on ice conditions in the Baltic Sea: The wavelet approach. Journal of Geophysical Research: Atmospheres. 2003 11 16;108(D21). http://citeseerx.ist.psu.edu/viewdoc/download?doi = 10.1.1.728.5758&rep = rep1&type = pdf.

[26] Torrence C, Compo GP. A practical guide to wavelet analysis. Bulletin of the American Meteorological society. 1998 1;79(1):61–78. https://psl.noaa.gov/people/gilbert.p.compo/Torrence_compo1998.pdf.

[27] Barragan V, Olivas S, Keim P, Pearson T. Critical knowledge gaps in our understanding of environmental cycling and transmission of Leptospira spp. Applied and environmental microbiology. 2017 10 1;83(19). https://www.ncbi.nlm.nih.gov/pmc/articles/PMC5601346/. 10.1128/AEM.01190-17 28754706 PMC5601346

[28] Kumar KV, Lall C, Raj RV, Vedhagiri K, Maile A, Muruganandam N, et al Detection of pathogenic Leptospira in the environment and its association with antagonistic Pseudomonas spp. and rainy season. bioRxiv. 2020 1 1 10.1101/2020.05.03.074963.

[29] Casanovas-Massana A, Pedra GG, Wunder EA, Diggle PJ, Begon M, Ko AI. Quantification of Leptospira interrogans survival in soil and water microcosms. Applied and environmental microbiology. 2018 7 1;84(13). https://www.ncbi.nlm.nih.gov/pmc/articles/PMC6007094/. 10.1128/AEM.00507-18 29703737 PMC6007094

[30] Ellis WA. Animal leptospirosis InLeptospira and leptospirosis 2015 (pp. 99–137). Springer, Berlin, Heidelberg https://pubmed.ncbi.nlm.nih.gov/25388134/.

[31] Thiermann AB. The Norway rat as a selective chronic carrier of Leptospira icterohaemorrhagiae. Journal of wildlife diseases. 1981 1;17(1):39–43. https://pubmed.ncbi.nlm.nih.gov/7253100/. 10.7589/0090-3558-17.1.39 7253100

[32] Agampodi SB, Dahanayaka NJ, Bandaranayaka AK, Perera M, Priyankara S, Weerawansa P, et al Regional differences of leptospirosis in Sri Lanka: observations from a flood-associated outbreak in 2011. PLoS Negl Trop Dis. 2014 1 16;8(1):e2626 https://www.ncbi.nlm.nih.gov/pmc/articles/PMC3894175/. 10.1371/journal.pntd.0002626 24454971 PMC3894175

[33] Bandara JM, Wijewardena HV, Liyanege J, Upul MA, Bandara JM. Chronic renal failure in Sri Lanka caused by elevated dietary cadmium: Trojan horse of the green revolution. Toxicology Letters. 2010 9 15;198(1):33–9. https://www.ncbi.nlm.nih.gov/pmc/articles/PMC7127468/. 10.1016/j.toxlet.2010.04.016 20430069 PMC7127468

[34] Director General of Sri Lanka Disaster Management Center, Asian Conference on Disaster Reduction, June 13–15, 2011, Cinnamon Grand Hotel Colombo Sri Lanka. Presentation ‘Recent Floods in Sri Lanka’ by the Director General of Sri Lanka Disaster Management Center. https://www.adrc.asia/acdr/2011colombo/documents/01_Recent%20Floods%20in%20Sri%20Lanka%20-%2010.06.2011.pdf.

[35] Epidemiology Unit, Ministry of Health- Sri Lanka. New Challenges in Controlling Leptospirosis. Weekly Epidemiological Report Vol38,No.08. 2011—Epidemiology Unit, Ministry of Health- Sri Lanka.

[36] Reason J. Human error: models and management. Bmj. 2000 3 18;320(7237):768–70. https://www.ncbi.nlm.nih.gov/pmc/articles/PMC1070929/. 10.1136/bmj.320.7237.768 10720363 PMC1117770

[37] Epidemiology Unit, Ministry of Health- Sri Lanka. New Challenges in Controlling Leptospirosis. Weekly Epidemiological Report Vol38,No.08. 2011. Epidemiology Unit, Ministry of Health- Sri Lanka.

[38] Naotunna C, Agampodi SB, Agampodi TC. Etiological agents causing leptospirosis in Sri Lanka: A review. Asian Pacific journal of tropical medicine. 2016 4 1;9(4):390–4. https://pubmed.ncbi.nlm.nih.gov/27086159/. 10.1016/j.apjtm.2016.03.009 27086159

[39] Ehelepola ND, Wijesinghe WM. An analysis of a dengue outbreak at a large hospital and Epidemiological evidence for nosocomial dengue. Journal of tropical medicine. 2018 6 26;2018 https://www.ncbi.nlm.nih.gov/pmc/articles/PMC6038582/. 10.1155/2018/9579086 PMC6038582 30046313

[40] Tun MM, Muthugala R, Nabeshima T, Rajamanthri L, Jayawardana D, Attanayake S, et al Unusual, neurological and severe dengue manifestations during the outbreak in Sri Lanka, 2017. Journal of Clinical Virology. 2020 2 27:104304 https://pubmed.ncbi.nlm.nih.gov/32145478/. 10.1016/j.jcv.2020.104304 32145478

[41] Gamage CD, Koizumi N, Perera AC, Muto M, Nwafor‐Okoli C, Ranasinghe S, et al Carrier Status of Leptospirosis Among Cattle in Sri Lanka: A Zoonotic Threat to Public Health. Transboundary and emerging diseases. 2014 Feb;61(1):91–6. https://pubmed.ncbi.nlm.nih.gov/22998409/. 10.1111/tbed.12014 22998409

[42] Storck CH, Postic D, Lamaury IP, Perez JM. Changes in epidemiology of leptospirosis in 2003–2004, a two El Nino Southern Oscillation period, Guadeloupe archipelago, French West Indies. Epidemiology & Infection. 2008 10;136(10):1407–15. https://www.ncbi.nlm.nih.gov/pmc/articles/PMC2870739/. 10.1017/S0950268807000052 18096102 PMC2870739

[43] Bharti AR, Nally JE, Ricaldi JN, Matthias MA, Diaz MM, Lovett MA, et al Leptospirosis: a zoonotic disease of global importance. The Lancet infectious diseases. 2003 12 1;3(12):757–71. https://pubmed.ncbi.nlm.nih.gov/14652202/. 10.1016/s1473-3099(03)00830-2 14652202

[44] Premdas AK, Areekal B, Sukumaran ST, Kandi AR. Trend of leptospirosis and its association with meteorological factors in Thrissur district, Kerala. International Journal of Community Medicine and Public Health. 2019 11;6(11):4857 10.18203/2394-6040.ijcmph20195068.

[45] Desvars A, Jégo S, Chiroleu F, Bourhy P, Cardinale E, Michault A. Seasonality of human leptospirosis in Reunion Island (Indian Ocean) and its association with meteorological data. PloS one. 2011 5 31;6(5):e20377 https://www.ncbi.nlm.nih.gov/pmc/articles/PMC3105052/. 10.1371/journal.pone.0020377 21655257 PMC3105052

[46] Joshi YP, Kim EH, Cheong HK. The influence of climatic factors on the development of hemorrhagic fever with renal syndrome and leptospirosis during the peak season in Korea: an ecologic study. BMC infectious diseases. 2017 12;17(1):1–1. https://www.ncbi.nlm.nih.gov/pmc/articles/PMC5463320/. 10.1186/s12879-016-2122-x 28592316 PMC5463320

[47] Chadsuthi S, Wong-Ekkabut J, Triampo W, Doungchawee G, Triampo D. Comparison of the effects of UV-A radiation on Leptospira interrogan serovar Bataviae, Canicola and Pomona. African Journal of Biotechnology. 2010;9(21):3196–206. https://academicjournals.org/journal/AJB/article-full-text-pdf/D6AFAB123981.

[48] Wikipedia. Climate of Seoul.Statistics 2020 https://en.wikipedia.org/wiki/Climate_of_Seoul#cite_note-12.

[49] Arias-Monsalve C, Builes-Jaramillo A. Impact of El Niño-Southern oscillation on human leptospirosis in Colombia at different spatial scales. The Journal of Infection in Developing Countries. 2019 12 31;13(12):1108–16. https://pubmed.ncbi.nlm.nih.gov/32088698/. 10.3855/jidc.11702 32088698

[50] Jayakody P.M. The Influence of La Nina on Sri Lanka Rainfall. Sri Lanka Journal of Meteorology, Volume1 2015 http://www.meteo.gov.lk/images/sljom/preethika.pdf.

[51] Yapa A, Ratnavira G.b 1st edition 2013 Field Ornithology Group of Sri Lanka, Department of Zoology, University of Colombo, Colombo, Sri Lanka.

[52] Andersen-Ranberg EU, Pipper C, Jensen PM. Global patterns of Leptospira prevalence in vertebrate reservoir hosts. Journal of wildlife diseases. 2016 7;52(3):468–77. https://pubmed.ncbi.nlm.nih.gov/27187029/. 10.7589/2014-10-245 27187029

[53] Zinsstag J, Utzinger J, Probst-Hensch N, Shan L, Zhou XN. Towards integrated surveillance-response systems for the prevention of future pandemics. Infectious diseases of poverty. 2020 12;9(1):1–6. https://www.ncbi.nlm.nih.gov/pmc/articles/PMC7539270/. 10.1186/s40249-019-0617-6 33028426 PMC7539270

[54] Punyawardena R., Dissanaike T and Mallawatantri A. Spatial Variation of Climate Change Induced Vulnerability in Sri Lanka: An Analysis of the Components of Vulnerability at District Level.2013 Department of Agriculture, Peradeniya, Sri Lanka.

[55] Abd Rahim MA, Zaki AM, Atil A, Azme MH, Him NA, Rahim SS, et al Effectiveness of Antibiotic Prophylaxis for Leptospirosis among Adults: A Systematic Review. Malaysian Journal of Applied Sciences. 2018 12 30;3(2):46–56. https://journal.unisza.edu.my/myjas/index.php/myjas/article/view/144.

[56] Fonseka CL, Vidanapathirana BN, de Silva CM, Athukorala AA, Goonawardena PR, Karunathilake AP, et al Doxycycline Usage for Prevention of Leptospirosis among Farmers and Reasons for Failure to Use Chemoprophylaxis: A Descriptive Study from Southern Sri Lanka. Journal of Tropical Medicine. 2019 3 3;2019. https://www.ncbi.nlm.nih.gov/pmc/articles/PMC6420970/. 10.1155/2019/2917154 30941181 PMC6420970

[57] Golden I, Bracho G. A reevaluation of the effectiveness of homoeoprophylaxis against leptospirosis in Cuba in 2007 and 2008. Journal of evidence-based complementary & alternative medicine. 2014 7;19(3):155–60. https://pubmed.ncbi.nlm.nih.gov/24647096/. 10.1177/2156587214525402 24647096

